# ^1^H-NMR-based urine metabolomics of prostate cancer and benign prostatic hyperplasia

**DOI:** 10.1016/j.heliyon.2024.e28949

**Published:** 2024-03-31

**Authors:** Mohammed Zniber, Tarja Lamminen, Pekka Taimen, Peter J. Boström, Tan-Phat Huynh

**Affiliations:** a Laboratory of Molecular Science and Engineering, Åbo Akademi University, Turku, Finland; b Department of Urology, University of Turku and Turku University Hospital, Turku, Finland; c Institute of Biomedicine and FICAN West Cancer Centre, University of Turku and Department of Pathology, Turku University Hospital, Turku, Finland

**Keywords:** NMR, Metabolomics, Urine, Prostate cancer, Chemometrics

## Abstract

**Background:**

Prostate cancer (PCa) and benign prostatic hyperplasia (BPH) are prevalent conditions affecting a significant portion of the male population, particularly with advancing age. Traditional diagnostic methods, such as digital rectal examination (DRE) and prostate-specific antigen (PSA) tests, have limitations in specificity and sensitivity, leading to potential overdiagnosis and unnecessary biopsies.

**Significance:**

This study explores the effectiveness of ^1^H NMR urine metabolomics in distinguishing PCa from BPH and in differentiating various PCa grades, presenting a non-invasive diagnostic alternative with the potential to enhance early detection and patient-specific treatment strategies.

**Results:**

The study demonstrated the capability of ^1^H NMR urine metabolomics in detecting distinct metabolic profiles between PCa and BPH, as well as among different Gleason grade groups. Notably, this method surpassed the PSA test in distinguishing PCa from BPH. Untargeted metabolomics analysis also revealed several metabolites with varying relative concentrations between PCa and BPH cases, suggesting potential biomarkers for these conditions.

## Highlights

• We present a metabolomics study using ^1^H NMR to differentiate prostate cancer from benign prostatic hyperplasia.
• Urine NMR profiles effectively distinguish between prostate cancer and benign prostatic hyperplasia.
• ^1^H NMR outperforms the PSA test in terms of specificity, sensitivity, accuracy, and predictive ability.
• Potential utility of ^1^H NMR in grading prostate cancer based on Gleason grade group.
• Identification of six statistically significant metabolites contributing to this discrimination.

## Graphical abstract

**Figure:**
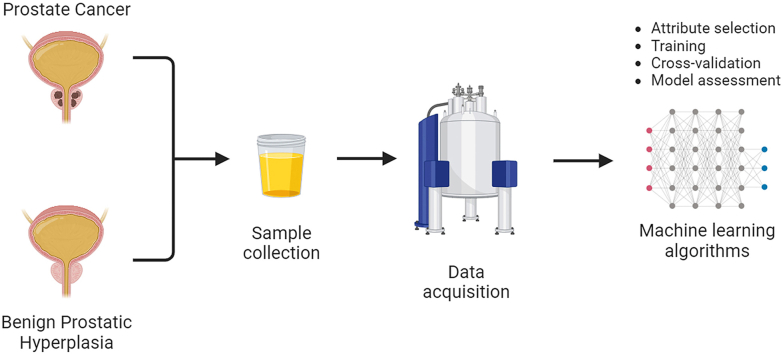

## 1 Introduction

Prostate cancer (PCa) is the fourth leading cause of death and the second most widely diagnosed cancer type among men. In 2020, PCa accounted for approximately 374 000 deaths and 1.42 million new cases [1] whereas benign prostatic hyperplasia (BPH) impacts 62% of men aged >50 years old [[2], [3], [4]]. PCa and BPH are major health problems and the incidences of both diseases are expected to increase due to aging of the population [5]. With similarities and frequent coexistence between these two pathological conditions, the question of their relation is raised. Based on epidemiological studies the incidence of PCa and BPH is tightly correlated with age. Both are also hormone dependent [6], for instance, androgens are essential for normal prostate growth as well as the development of PCa and BPH. In brief, 5-α reductase type 1 and 2, are two isozymes involved in converting testosterone to dihydrotestosterone (DHT), which binds to androgen receptors and promotes cellular differentiation and proliferation within the prostate. The abnormal growth of the prostate is considered to involve the disruption of DHT-supported homeostasis between cell death and cell proliferation resulting in an inhibition of apoptotic processes and predomination of proliferative processes [5,[7], [8], [9]].

The most common diagnostic methods of PCa include digital rectal examination (DRE), prostate-specific-antigen (PSA), transrectal ultrasound (TRUS), magnetic resonance imaging (MRI) and prostate biopsy. Among them, PSA test is widely used method for screening purposes to estimate the risk of PCa. However, it suffers from various disadvantages including high false positivity and poor specificity of PSA for detecting cancer which leads to unnecessary prostate cancer biopsies [[10], [11], [12]].

In contrast to the abovementioned diagnostic methods, nuclear magnetic resonance (NMR) spectroscopy-based urine metabolomics could be useful in discriminating PCa and BPH. For instance, NMR spectroscopy is non-invasive, non-destructive, and does not require chromatographic separation, sample treatment, or chemical derivatization. Moreover, NMR is highly automatable, rapid, and reproducible; thus large-scale metabolomics studies are more feasible with NMR compared to other metabolomics platforms. For instance, compared to NMR, GC-MS and LC-MS can only process 20 to 30 percent of samples over the course of a 24-h period. This is due to longer chromatographic runs, manual sample preparation, and lengthy computer processing [13]. Furthermore, NMR is capable of detecting compounds that are less detectable in LC-MS such as sugars, organic acids, alcohols, polyols and other highly polar compounds [14]. NMR can also be used for quantitative analysis and enables the identification of several metabolites in a single experiment [15]. Recent studies have revealed the potential of ^1^H-NMR-based metabolomics in cancer diagnosis such as colorectal cancer [[16], [17], [18]], breast cancer [19,20] and other types of cancer [[21], [22], [23]]. The literature regarding PCa and BPH studies based on urine and NMR metabolomics is relatively scarce [12,[24], [25], [26], [27], [28]]. Additionally, most studies have focused on the analysis of tissue samples [[29], [30], [31], [32], [33], [34]] or serum/plasma [27,28,[35], [36], [37], [38], [39]]. Despite its advantages, NMR faces significant limitations, including poor sensitivity that requires metabolite concentrations of at least 1 μM for detection, limiting its ability to identify low-abundance metabolites essential for comprehensive metabolomic studies. Like MS, NMR cannot detect all metabolites, necessitating the combined use of NMR and MS to enhance metabolome coverage and metabolite identification accuracy. NMR is also prone to variability in spectral data due to changes in sample conditions like pH, temperature, ionic strength, and composition, complicating data interpretation and comparison. However, advancements in NMR technology are improving its accessibility, affordability, and clinical application compatibility [13,40].

In this study, we have investigated the metabolic differences between PCa and BPH using two different approaches of NMR spectroscopy: binning or bucketing and relative quantifications. Binned spectra were explored using two unsupervised techniques namely principal component analysis (PCA) and unsupervised random forest (URF). A classification model was built based on supervised random forest (RF) for the discrimination of the two studied groups. On the other hand, peak identification was used to measure relative quantifications of metabolites and determine the most significantly different ones between PCa and BPH.

## 2 Materials and methods

### 2.1 Study design and participants

The study protocol was approved by the Ethics Committee of the Hospital District of Southwest Finland (3/1801/2013). The volunteers enrolled for the study due to a clinical suspicion of PCa based on either elevated PSA levels or physical examination (palpation). A total of 81 patients included in this study, were encoded, comprising 40 pathologically confirmed PCa patients and 41 BPH patients. Individual donors could not be identified from the samples. Clinical characteristics of the individuals included in the study are shown in Table 1. All the urine samples were obtained perioperatively from patients undergoing transurethral resection of the prostate (TURP) for BPH or robot-assisted laparoscopic prostatectomy (RALP) for PCa at Turku University Hospital (TYKS) between 2021 and 2022.

**Table 1:** Clinical data of the individuals included in the study.

	BPH group (n = 41) (Median, range)	PCa group (n = 40) (Median, range)
Age (years)	74.4 (66.2–85.2)	66.2 (48.1–72.3)
BMI (kg m^−2^)	25.2 (18.7–30.7)	27.1 (21.3–31.3)
Preop PSA (ng mL^−1^)	3.4 (0.025–35)	7.9 (2.4–30)
Number of cores	N/A	14 (3–31)
Positive cores	N/A	0 (0–2)
Grade group	N/A	3 (2–5)

Explicitly, urine samples were taken using an indwelling catheter placed routinely for patients undergoing a urological operation. Urine collection did not include any additional instruments or surgical procedures. All collected samples were stored in a – 80 °C.

### 2.2 ^1^H NMR spectroscopy

#### 2.2.1 Reagents

Sodium (3-trimethylsilyl)-2,2,3,3-tetradeuteriopropionate (TSP), sodium chloride (NaCl), deuterium oxide (D_2_O), sodium phosphate monobasic (NaH_2_PO_4_) and sodium phosphate dibasic (Na_2_HPO_4_) were purchased from Sigma-Aldrich.

#### 2.2.2 Measurement

Aliquots of 450 μL human urine were mixed with 50 μL of D_2_O containing 1 mM of TSP, 0.2 M of Na_2_HPO_4_ | 0.04 M of NaH_2_PO_4_ (pH 7.4) and 0.8 w/v% of NaCl to a total volume of 500 μL in Eppendorf tubes. The pH adjustment of all urine samples needed to be done before conducting any measurements to ensure consistency and reliability in our NMR experiments. This step is crucial as the pH can significantly affect the outcome of NMR spectroscopy by influencing the chemical shift and, consequently, the interpretation of the spectra [41]. The samples were then centrifuged at 3000 rpm for 5 min and transferred to clean 5 mm NMR tubes using fine Pasteur pipettes. Finally, the tubes were capped and labelled prior to NMR acquisition. NMR experiments were carried out using a 600 MHz NMR spectrometer (AVANCE III, Bruker, Germany) equipped with a liquid nitrogen cooled Prodigy TCI (inverted CryoProbe) at 298 K.

### 2.3 Data preprocessing and analysis

Raw FIDs (free induction decay) were preprocessed using the R statistical software package PepsNMR [42]. Subsequently, binned spectra were analyzed, employing techniques such as PCA and URF, to unveil clustering patterns and detect outliers within the two studied groups. Discriminant analysis was performed using RF to distinguish between the two studied groups. Metabolite identification and relative quantifications were carried out using ASICS [43]. Finally, statistical differences between PCa and BPH were assessed using the Kruskal-Wallis test.

## 3 Results

### 3.1 Data preprocessing

**Fig. 1:**
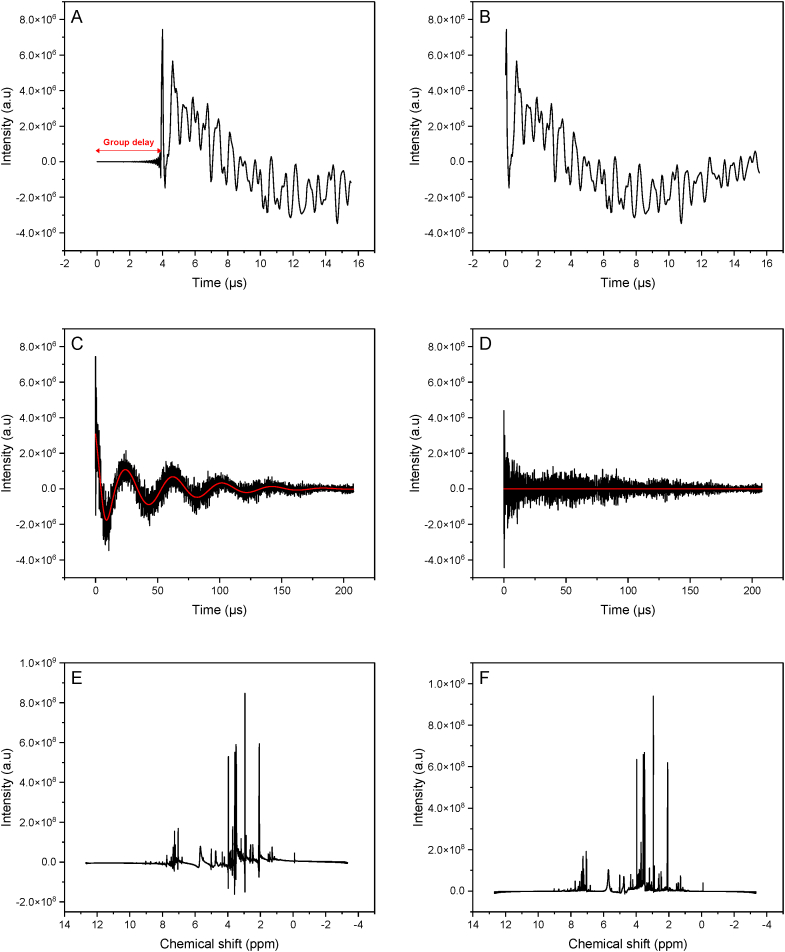
Zoomed FID with group delay (A) and after group delay removal (B). FID spectrum with (C) and without solvent residuals signal (D). NMR spectrum after Fourier Transform (E) and after zero-order phase correction (F).

Initially, the raw Bruker files were imported into R resulting into two data frames: the complex FID signal matrix and the Bruker acquisition. Fig. S1 provides a visual representation of the real of the FID. Fig. 1A and B presents an NMR spectrum before and after correcting for group delay or death time. The FID indicates a death time of about 4 μs, with negligible values before the start of the standard FID. Prior to group delay correction, the Fourier transform spectrum exhibits baseline fluctuations similar to the convolution with a sinc function centered in the spectrum. This can be explained by the Fourier transform's time shift theorem, which suggests that shifting the time domain signal by n points results in the frequency domain spectrum being the standard spectrum (unshifted FID) multiplied by exp(-i2*π*w*n) [42]. Hence, these baseline wiggles due to a first-order phase shift introduced by the group delay can be rectified by subtracting the group delay from the FID. Next, the FIDs are processed using a Whittaker smoother to remove the variability of solvent (water) residuals in the spectrum, which can mask informative signals of other interesting compounds. Fig. 1C and D shows the estimated solvent residuals signal as well as the FID signal before and after its removal. Subsequently, the FIDs are converted into spectra in the frequency domain by Fourier Transform (Fig. 1E). This figure shows that the spectra present zero-order phase error of a certain angle, which is corrected by automatically finding an optimal φ_0_ which shifts the spectrum into its pure absorptive mode (Fig. 1F).

**Fig. 2:**
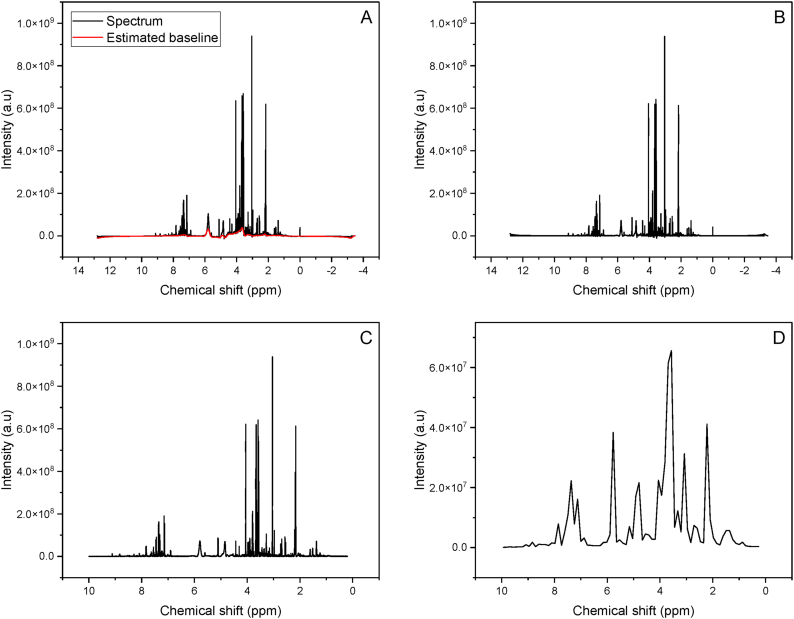
NMR spectrum before (A) and after baseline correction (B). NMR spectrum before (C) and after bucketing (D).

Consequently, the NMR spectra are referenced with TSP (Fig. S2), a known reference compound whose chemical shift is set to 0 ppm by convention. A baseline removal was calculated and removed from the spectra as shown in Fig. 2A and B.

Negative values from prior steps are set to zero as they lack meaningful interpretation. Next, spectra are globally aligned to minimize misalignment between identical peaks across different spectra. Negative values from prior steps are set to zero as they lack meaningful interpretation. Subsequently, spectra are globally aligned to minimize misalignment between identical peaks across different spectra. Finally, the high dimensions of the spectra is reduced using rectangular bucketing (Fig. 2C and D).

Region removal was applied to cut off the region (4.5–6.1 ppm) where the water resonance residuals, urea, and maleic acid are located. This region is influenced by the removal of the water resonance using presaturation. Finally, each spectrum was normalized by quantile normalization which attempts to achieve the same distribution of feature intensities across all spectra [44].

### 3.2 Analysis on buckets

#### 3.2.1 Principal component analysis

**Fig. 3:**
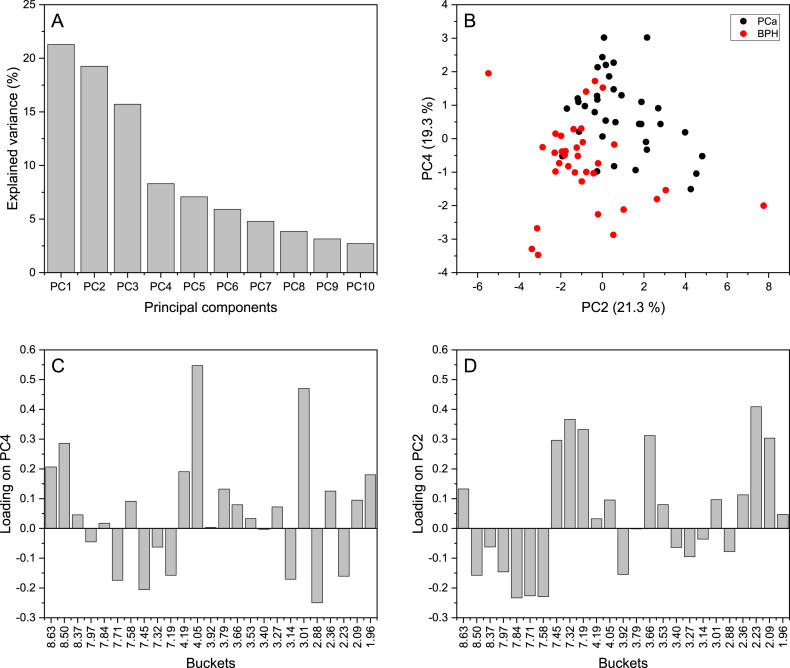
Percentage of variance explained by PCs obtained from NMR binned spectra (A) and (B) scatter plot of PC2 versus PC4 (B); loading plots for PC4 (C) and PC2 (D)

The binned spectra were mean-centered, and any columns with all zeros were excluded. PCA was subsequently employed to explore potential associations and patterns in the data. Fig. 3A presents the first ten principal components (PCs), which collectively explain 90% of the total variance observed. In Fig. 3B, the score plot between PC1 and PC4 reveals that BPH samples tend to cluster with negative values of PC4, while PCa samples cluster with positive values of PC4. Additionally, PC2 exhibited slight separation of samples. This separation can be explained by the loadings illustrated in Fig. 3C and D, for instance, buckets located around 1.96, 2.09, 2.36, 3.01, 3.79, 4.05, 4.19, 8.50, and 8.63 ppm are related to the PCa patients while 2.23, 2.88, 3.14, 7.19, 7.45, and 7.71 ppm are related to BPH individuals. Additionally, loading of PC2 indicates that buckets around 7.58, 7.71, 7.84, 7.97, and 8.50 ppm are slightly related to the BPH group while 7.45, 7.32, 7.19, 3.66, 2.23, 2.09 ppm are slightly linked to the PCa group.

#### 3.2.2 Random forest

The dataset was divided into training and testing sets (test size of 25%). RF classifier was then trained on the training dataset using repeated cross validation approach with a number of repetitions of one hundred and number of splits of five. Next, the testing set was used to predict the output classes and evaluate the performance of the model using the confusion matrix and receiver operating characteristic (ROC) curve.

Table 2, Table 3 display the average confusion matrix and classification report of the RF model. The misclassification rates were around 6.8% and 5% for cross validation (CV) and testing set, respectively, and were calculated as the number of all incorrect predictions divided by the total number of predictions. In addition, the average recalls were 93% and 95%, precisions were 96% and 93% and accuracies were 93% and 95% in CV and testing indicating that the model generalizes on unseen data.

**Table 2:** Confusion matrix of RF obtained in cross validation.

		Predicted	
	Actual	BPH	PCa
**Training set**	BPH	22	1
	PCa	2	22
**Testing set**	BPH	10	0
	PCa	1	10

**Table 3:** Classification report calculated from Table 1.

		Precision	Recall	Accuracy	F1-score
**Training set (Cross-validation)**	BPH	0.92	0.93	0.93	0.93
	PCa	0.93	0.92	0.93	0.93
**Testing set**	BPH	1.00	0.90	0.95	0.95
	PCa	0.92	1.00	0.95	0.96

#### 3.2.3 Unsupervised random forest

**Fig. 4:**
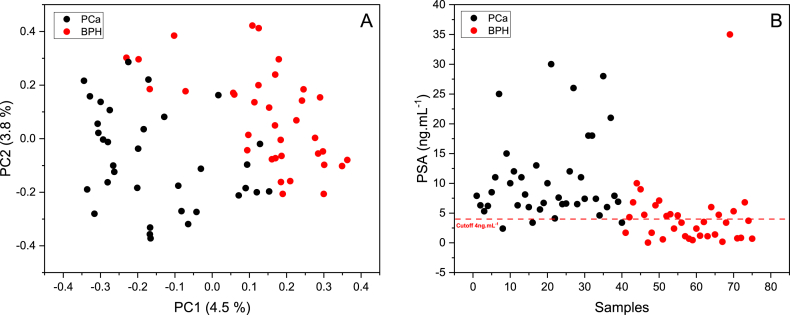
PCA score plot obtained from URF proximity matrix (A); Scatter plot showing patients, their PSA level and the cutoff used to discriminate PCa and BPH (B)

In order to explain the efficiency of the RF classification model, the original data was visualized using a score plot based on PCA performed on the proximity matrix obtained from URF [45]. The proximity matrix symbolizes the similarities between observations, for instance, a large proximity value implies similarity while a small value indicates dissimilarity. Fig. 4A displays a score plot of PC1 versus PC2 calculated from the URF proximity matrix; two distinct groups appear corresponding the two pathological conditions BPH and PCa. The dissimilarities between the two groups are quite clear except for some samples that look a bit similar supporting the abovementioned results of RF in discriminating between BPH and PCa.

#### 3.2.4 Prostate-specific-antigen test

PSA is a protein produced by the prostate gland and commonly measured as a screening test for prostate cancer. PSA levels are measured with a simple blood test, measured in nanograms of PSA per milliliter (ng mL^−1^) of blood. A higher PSA level may indicate an increased risk of prostate cancer, but it is not a definitive diagnosis. Other factors, such as age, family history, and medical history, should also be considered when interpreting the results of a PSA test. Fig. 4B represents BPH and PCa samples and their PSA levels. A cutoff point of ng mL^−1^ or higher was used to decide if a person might have PCa and lower in the case of BPH. It can be seen that most of the PCa samples were classified correctly (above the cutoff) unlike the BPH samples where half of the samples were misclassified as PCa. Hence, high false positivity and poor specificity of PSA for discriminating PCa from BPH which results in unnecessary biopsies.

#### 3.2.5 Comparison of ^1^H NMR and PSA

**Fig. 5:**
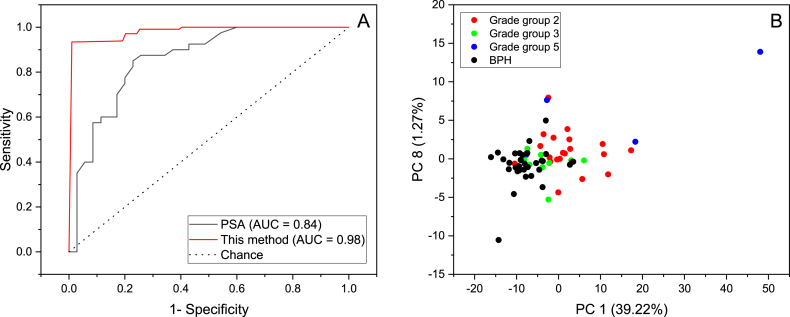
ROC curve of PSA and the Random Forest model (A), PCA score plot of patients diagnosed with BPH and PCa (Grade Group 2, 3 and 5) (B)

Table 4 represents the confusion matrix calculated using the PSA cutoff of 4 ng/mL. It shows that the PCa samples were mostly predicted correctly unlike BPH samples. This can be seen from Table 5 by the higher and lower precision and recall obtained for PCa and BPH, respectively. By comparing the results obtained from ^1^H NMR and PSA (Table 3 and Table 5), it can be clearly seen that NMR outperforms PSA in the prediction of PCa samples and especially in predicting BPH samples as PSA is not very sensitive to BPH. Fig. 5A illustrates the ROC curve for both PSA and RF model, along with their respective Area Under the Curve (AUC) values. The AUC of PSA is 0.84, which demonstrates its diagnostic potential. In comparison, the RF model achieved an even higher AUC of 0.98, highlighting its superior predictive ability. Meanwhile, the black dotted line represents the AUC of a random classifier, which is 0.5, indicating no discriminatory power between the two conditions.

**Table 4:** Confusion matrix of PSA test.

Actual	Predicted	
	BPH	PCa
BPH	37	15
PCa	3	20

**Table 5:** Classification report calculated from Table 3.

	Precision	Recall	Accuracy	F1-score
BPH	0.87	0.57	0.76	0.80
PCa	0.71	0.93	0.76	0.69

#### 3.2.6 Gleason score and grade group

The Gleason grading system, employed by pathologists to assess prostate cancer aggressiveness, assigns primary and secondary grade patterns scores (1–5) that are combined to generate Gleason scores (2–10). More recently, ISUP grade groups (GG1-GG5) have been introduced [46] and adopted, and they offer predictive value for recurrence-free progression in both biopsies and RP specimens following prostate cancer treatments [47]. The preprocessed spectra were employed to explore the potential of ^1^H NMR's in separating between patients diagnosed with BPH and PCa based on their Gleason grade group. While ^1^H NMR exhibited promise in this regard (Fig. 5B), further confirmation necessitates a larger sample set.

### 3.3 Analysis on relative concentrations

**Fig. 6:**
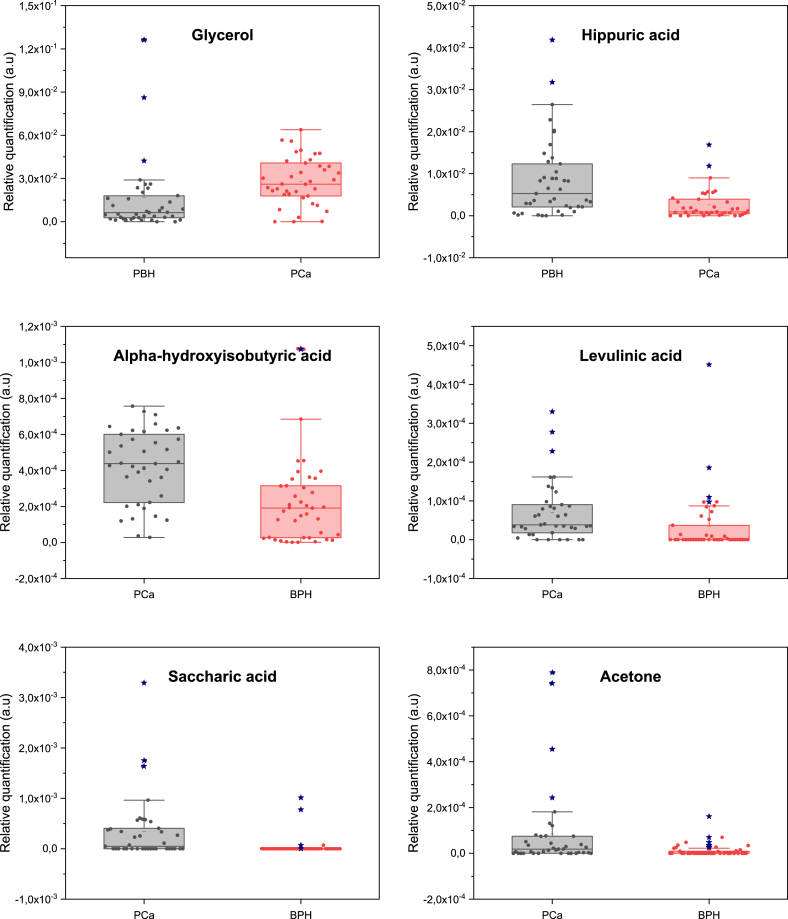
Relative concentrations of the six high statistically significant metabolites: glycerol, hippuric Acid, alpha-hydroxyisobutyric acid, levulinic acid, saccharic acid, and acetone in PCa vs. BPH.

**Fig. 7:**
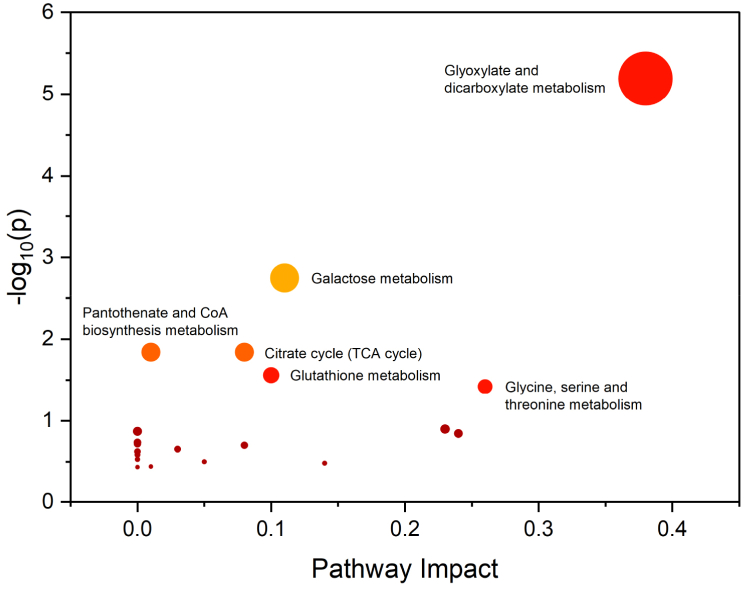
Pathway analysis conducted with MetaboAnalyst 6.0 identified six significant metabolic pathways.

Untargeted metabolomics identified 183 metabolites using ASICS, while metabolites with over 50% missing values were excluded. Kruskal-Wallis test revealed that forty-seven metabolites showed significant differences (p-value <0.05) between the two studied groups (Table S1). Moreover, six metabolites illustrated in Fig. 6, exhibited high statistical significance (p-value <0.001). These six metabolites showed a significant increase in relative concentrations, while relative concentrations of hippuric acid (HA) decreased (from BPH to PCa). Similar studies [36,48] showed a decreasing level of HA in urine and serum. However, the levels of hippurate can be influenced by other factors including physical conditions, emotional states, dietary habits, psychiatric disorders, and the presence of certain diseases [49]. Organic acids including acetic acid, pantothenic, benzoic, formic, malonic, pyroglutamic, succinic, 3-hydroxyisovaleric, isocitric, and 2-aminoadipic acid were also different between PCa and BPH. For instance, acetic acid, isocitric acid were down-regulated in PCa compared to BPH and accords with [48] except that they investigated their differences in PCa and HC. Our results also revealed a significant increase of 3-hydroxyisovaleric acid in PCa which is in agreement with [50] in prostate cancer cells compared to normal cells. Similarly, succinic acid and malonic acid were also increased in PCa compared to BPH, but, succinic was down-regulated in PCa versus HC [48]. 2-aminoadipic acid showed lower concentrations in PCa, unlike its elevated concentration in malignant tissue samples compared to non-malignant ones [51,52]. Benzoic acid was also decreased in patients diagnosed with PCa and accords with [48] which also found to be decreasing in ovarian cancer compared to ovarian benign tumor [53]. Similarly, pantothenic acid was also down-regulated in PCa, but it was found to be increased in PCa compared to BPH tissue extracts [52] and correlated with Gleason score which indicates its potential as a prostate cancer biomarker [54]. Pyroglutamic acid was decreased in PCa compared to BPH samples, however, it was found to be increasing in plasma of PCa samples [55]. Other organic acids were also significantly different such as 4-aminohippuric acid and glyoxylic acid, however, their role in prostate cancer have never been reported before. The amounts of lactose and citrulline were also down-regulated in PCa compared to BPH similar to Ref. [48] where the two metabolites were decreased in PCa compared with HC. Also, tryptophan decreased in PCa and are in accordance with [48,55] in urine and plasma, respectively. The decreased levels of glycine in urine of PCa compared to BPH was also observed and agrees with recent studies [48,56]. The levels of uracil were decreased in PCa, unlike other studies that reported its increased levels in BPH and PCa serum samples compared to HC group [37] as well as in urine samples of PCa and BPH [57]. A significant increase of creatinine in PCa was also observed and is in agreement with previous studies in filtered-serum of PCa compared with BPH and HC [37]. Indoxyl sulfate was also found to be decreased in urine samples of PCa and accords well with [58]. Moreover, this metabolites was also detected and quantified in serum samples, however, its concentration was higher in PCa compared to HC [59]. Metabolic pathway analysis conducted through MetaboAnalyst has identified significant alterations (p < 0.05) in six pathways in PCa, highlighting substantial metabolic changes that are intricately linked with the disease's development and progression. These pathways include glyoxylate and dicarboxylate metabolism, galactose metabolism, pantothenate and CoA biosynthesis, citrate cycle (TCA cycle), glutathione metabolism, glycine, serine, and threonine metabolism (Fig. 7).

**Fig. 8:**
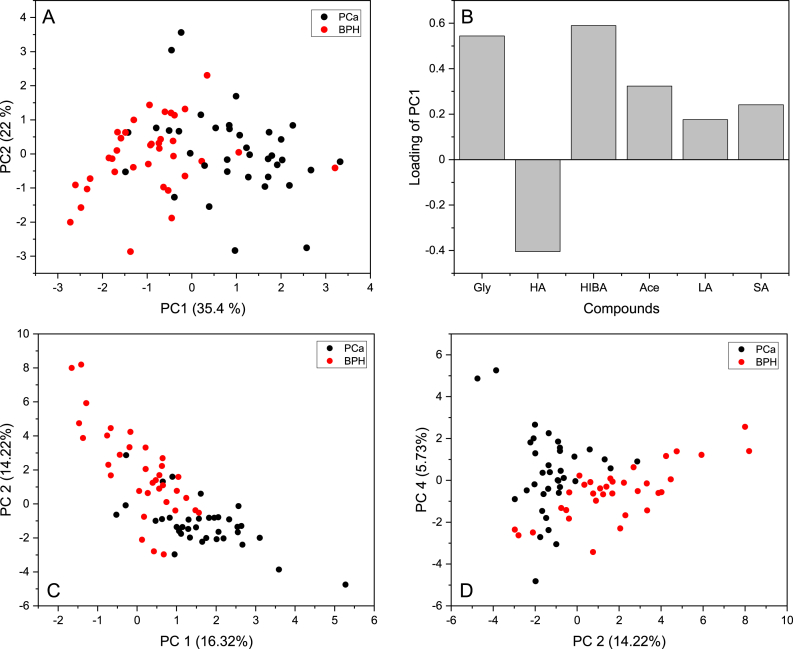
Score plot of PC1 vs PC2 and loading of PC1 calculated using only six highly different metabolites (A and B); Score plots of PC1 vs PC2 and PC2 vs PC4 obtained from all different metabolites (C and D)

Fig. 8A and B shows the score plot of the first two PCs obtained from the relative quantifications of the six highly different metabolites present in the two studied groups. It can be observed that the first component (PC1) is the main direction explaining the separation between PCa and BPH. This separation can be explained by the loading used to construct PC1 indicating that glycerol, acetone, levulinic acid, saccharic acid and alpha-hydroxyisobutyric acid are related to the PCa patients while hippuric acid is related to BPH individuals. Another PCA model was built using the whole statistically different metabolites between PCa and BPH. Fig. 8C and D displays score plots of PC1 vs PC2 and PC2 vs PC4 and indicate that PC2 is the most important principal component explaining the separation between the two studies groups. They also show that the model separates well the data compared to the one with only six compounds. This is due to the addition of other statistically different variables or compounds in the new model (Fig. S3 shows the loadings plot of PC1).

## 4 Discussion

The results presented in this study suggest that ^1^H NMR has a strong discriminatory power in separating PCa from BPH. The study utilized various techniques, such as PCA and URF, to demonstrate clear separation between the two groups. These findings are supported by both the score plots, which reveal distinct clusters for PCa and BPH, and the loading plots, which indicate the metabolites contributing to this separation. They also showed that ^1^H NMR outperforms the PSA test in terms of specificity, sensitivity, accuracy, and overall predictive ability, highlighting its potential as a more reliable diagnostic tool for distinguishing between PCa and BPH, potentially reducing unnecessary biopsies and improving patient care. This study also explored the potential of ^1^H NMR in distinguishing patients diagnosed with PCa and BPH based on their Gleason grade group and showed promising resulting on the utility of ^1^H NMR in grading PCa.

Untargeted metabolomics analysis identified several metabolites that exhibit significant differences between PCa and BPH. Notably, six metabolites— glycerol, acetone, levulinic acid, saccharic acid, alpha-Hydroxyisobutyric acid, and hippuric acid— emerged as highly statistically significant contributors to the differentiation between these two conditions. Furthermore, based on the relative concentrations of these six biomarkers, a PCA model was conducted, which further substantiated the ability to separate PCa from BPH.

The study points out the need for further research and validation, particularly with larger sample sizes. Additionally, exploring the metabolic pathways related to the identified metabolites and understanding their clinical relevance can provide valuable insights into the biology of PCa and BPH.

In conclusion, the findings of this study shed light on the potential of ^1^H-NMR-based urine metabolomics as a powerful tool for distinguishing between PCa and BPH. This approach offers improved screening compared to the traditional PSA test; however, further validation is needed.

## Ethical statement

The ethical statement is 3/1801/2013 given by Ethics Committee of the Hospital District of Southwest Finland. Participants are required to provide written informed consent.

## Funding

T-P H acknowledges the Liv och Hälsa Foundation, Academy of Finland (Grant No. 323240 and 331774), and the European Cooperation in Science and Technology (COST Action: CA17120) for financial support.

## Data availability

The data will be provided upon request due to its large size.

## CRediT authorship contribution statement

**Mohammed Zniber:** Writing – review & editing, Writing – original draft, Validation, Methodology, Formal analysis, Data curation, Conceptualization. **Tarja Lamminen:** Writing – review & editing, Resources, Methodology. **Pekka Taimen:** Writing – review & editing, Validation, Formal analysis. **Peter J. Boström:** Writing – review & editing, Resources, Formal analysis, Conceptualization. **Tan-Phat Huynh:** Writing – review & editing, Validation, Supervision, Resources, Funding acquisition, Formal analysis, Conceptualization.

## Declaration of competing interest

The authors declare that they have no known competing financial interests or personal relationships that could have appeared to influence the work reported in this paper.
