# Supplementary material for: 1H-NMR-based urine metabolomics of prostate cancer and benign prostatic hyperplasia
Source: Heliyon. 2024 Mar 31;10(7):e28949. doi: 10.1016/j.heliyon.2024.e28949 (PMC11015411; doi:10.1016/j.heliyon.2024.e28949)
Supplement: Multimedia component 1 [file mmc1.docx]

**Supplementary information file**

**^1^H-NMR based urine metabolomics of prostate cancer and benign prostatic hyperplasia**

Mohammed Zniber^a^, Tarja Lamminen^b^, Pekka Taimen^c^, Peter J. Boström^b^, Tan-Phat Huynh^a,^*

^a^ Laboratory of Molecular Science and Engineering, Åbo Akademi University, Turku, Finland

^b^ Department of Urology, University of Turku and Turku University Hospital, Turku, Finland

^c^ Department of Pathology, University of Turku and Turku University Hospital, Turku, Finland

**Corresponding author. Tel.: +358 504337295*

*Email:* [tan.huynh@abo.fi](mailto:tan.huynh@abo.fi)

Supplementary Figures S1-S3, Table 4

**Figure S1.** Raw FID (real part) of a urine sample.

**Figure S2.** Internal referencing of an NMR spectrum with TSP.

**Figure S3.** Loading of PC2 for the highly statistically different compounds between PCa and BPH.

**Table S1.** Significant metabolite biomarkers found in the urine of PCa and BPH groups.

| **No** | **HMDB ID** | **Metabolite** | **Regulation (PCa vs BPH)** | **Adjusted p-value** | **References** |
| --- | --- | --- | --- | --- | --- |
| 1 | HMDB0000131 | **Glycerol** | ↑ | 0.000 | N/A |
| 2 | HMDB0001659 | **Acetone** | ↑ | 0.000 | N/A |
| 3 | HMDB0000562 | Creatinine | ↑ | 0.017 | [39] |
| 4 | HMDB0000300 | Uracil | ↓ | 0.001 | [39,57] |
| 5 | HMDB0000714 | **Hippuric acid** | ↓ | 0.000 | [38,49] |
| 6 | HMDB0001867 | 4-Aminohippuric acid | ↓ | 0.007 | N/A |
| 7 | HMDB0000729 | **alpha-Hydroxyisobutyric acid** | ↑ | 0.000 | N/A |
| 8 | HMDB0000904 | L-Citrulline | ↑ | 0.018 | [49] |
| 9 | HMDB0000510 | 2-Aminoadipic acid | ↓ | 0.020 | [51,52] |
| 10 | HMDB0000123 | Glycine | ↑ | 0.037 | [49,56] |
| 11 | HMDB0000193 | Isocitric acid | ↓ | 0.001 | [49] |
| 12 | HMDB0000754 | 3-Hydroxyisovaleric acid | ↑ | 0.048 | [50] |
| 13 | HMDB0000720 | **Levulinic acid** | ↑ | 0.000 | N/A |
| 14 | HMDB0000119 | Glyoxylic acid | ↓ | 0.046 | N/A |
| 15 | HMDB0000254 | Succinic acid | ↑ | 0.027 | [49] |
| 16 | HMDB0000107 | Galactitol | ↓ | 0.011 | N/A |
| 17 | HMDB0000267 | Pyroglutamic acid | ↑ | 0.008 | [55] |
| 18 | HMDB0000691 | Malonic acid | ↑ | 0.050 | N/A |
| 19 | HMDB0000663 | **Saccaric acid** | ↑ | 0.000 | N/A |
| 20 | HMDB0000142 | Formic acid | ↓ | 0.022 | [60,61] |
| 21 | HMDB0001870 | Benzoic acid | ↓ | 0.001 | [49] [53] |
| 22 | HMDB0000210 | Pantothenic acid | ↓ | 0.004 | [52] [54] |
| 23 | HMDB0041627 | Lactose | ↓ | 0.031 | [49] |
| 24 | HMDB0000957 | Pyrocatechol | ↓ | 0.015 | N/A |
| 25 | HMDB0000929 | L-Tryptophan | ↓ | 0.012 | [49,55] |
| 26 | HMDB0000042 | Acetic acid | ↓ | 0.017 | [49] |
| 27 | HMDB0000682 | Indoxyl sulfate | ↓ | 0.037 | [58,59] |
